# Beneficial Effects of Viable and Heat-Inactivated *Lactobacillus rhamnosus* GG Administration on Oxidative Stress and Inflammation in Diet-Induced NAFLD in Rats

**DOI:** 10.3390/antiox12030717

**Published:** 2023-03-14

**Authors:** Laura Arellano-García, Jenifer Trepiana, J. Alfredo Martínez, María P. Portillo, Iñaki Milton-Laskibar

**Affiliations:** 1 Nutrition and Obesity Group, Department of Nutrition and Food Science, Faculty of Pharmacy and Lucio Lascaray Research Centre, University of the Basque Country (UPV/EHU), 01006 Vitoria-Gasteiz, Spain; 2 CIBERobn Physiopathology of Obesity and Nutrition, Institute of Health Carlos III, 28222 Madrid, Spain; 3 BIOARABA Health Research Institute, 01006 Vitoria-Gasteiz, Spain; 4 Precision Nutrition and Cardiometabolic Health, IMDEA-Food Institute (Madrid Institute for Advanced Studies), Campus of International Excellence (CEI) UAM+CSIC, Spanish National Research Council, 28049 Madrid, Spain

**Keywords:** NAFLD, liver steatosis, probiotics, parabiotics, *Lactobacillus rhamnosus* GG, oxidative stress, inflammation

## Abstract

Oxidative stress and inflammation are well-known triggers of NAFLD onset and progression. The aim of this study is to compare the potential benefits of a viable probiotic (*Lactobacillus rhamnosus* GG) and its parabiotic (heat-inactivated) on oxidative stress, inflammation, DNA damage and cell death pathways in the liver of rats featuring diet-induced NAFLD. The consumption of the steatotic diet led to increased final body and liver weights, higher hepatic triacylglycerol content, altered serum transaminase levels and enhanced oxidative and inflammatory status. Administration of the probiotic and the parabiotic partially prevented the body weight increase induced by the steatotic diet, whereas the probiotic caused more effective decreasing hepatic triglyceride content. Sharp but nonstatistically significant decreases in serum transaminase levels were also observed for both treatments. The reduction in antioxidant enzyme activities found in the nontreated animals fed the steatotic diet was partially prevented by both treatments (GPx activity). Similarly, the reductions in nonenzymatic antioxidant protection (GSH content) and total antioxidant capacity (ORAC) found in the nontreated rats were restored by the administration of both treatments. These results show that both viable and heat-inactivated *Lactobacillus rhamnosus* GG administration partially prevent steatotic diet-induced liver oxidative stress and inflammation induced in rats.

## 1. Introduction

Nonalcoholic fatty liver disease (NAFLD) prevails as the most common liver morbidity, representing the leading cause of liver-related morbidity and mortality [1] and becoming a major health menace worldwide. NAFLD encompasses a spectrum of hepatic conditions ranging from simple steatosis to more harmful stages including steatohepatitis (steatosis with inflammation and hepatocyte injury), fibrosis, cirrhosis, and hepatocellular carcinoma (HCC) [2]. NAFLD is diagnosed when hepatic lipid accounts for more than 5% of the liver weight without evidence of hepatocyte damage in the absence of excessive alcohol consumption [3,4]. Since the liver plays a key role on lipid and glucose metabolism, NAFLD is normally present in obese patients and represents a risk factor for the development of type 2 diabetes [5]. Indeed, the insulin resistance that is commonly present in obesity (the cornerstone for metabolic syndrome diagnosis) is considered the triggering event leading to excessive liver lipid accumulation (due to enhanced de novo lipogenesis and increased free fatty acid influx from white adipose tissue), resulting in hepatic steatosis [6]. As a result, the prevalence of NAFLD ranges from 60% to 95% among obese patients. Moreover, approximately 60% of the individuals who suffer from diabetes have also been diagnosed with NAFLD [7]. Given the impact of this liver disease in further metabolic impairments, NAFLD is commonly known as the hepatic manifestation of metabolic syndrome [8]. 

One of the common characteristics among patients who suffer from NAFLD is the consumption of unbalanced diets. In this regard, the current trend is to replace traditional diets for a Westernized dietary pattern, which is characterized by high contents of saturated fats and sugar. An excessive consumption of high fat foods and added sugars represents a major risk not only for NAFLD development, but also for its progression [8]. Among added sugars, fructose has gained special attention due its lipogenic capacity, acting both as a substrate and as an inducer of lipogenic pathways in the liver [9]. Moreover, its metabolization results in a temporary depletion of intracellular phosphate and adenosine triphosphate (ATP) levels, provoking a decrease in protein synthesis, an elevation of oxidative stress, and mitochondrial dysfunction [10].

Even though the dietary pattern is clearly associated with NAFLD development and progression, there is also a complex network of factors (environmental and genetic factors) that act jointly in the onset of NAFLD [6]. In this regard, a “multiple-hit” theory has been proposed to understand the underlying mechanisms leading to NAFLD [6]. This theory describes the different “hits” that may compromise the adipose tissue (AT), the gastrointestinal tract and the liver functionality, by way of the triangular interplay among these elements and its relationship with NAFLD progress [5]. Factors such as an unhealthy dietary pattern or obesity may derive from adipocyte hypertrophy, which causes alterations in AT, such as insulin resistance. This leads to an increased lipolysis, and thus a higher secretion of free fatty acids (FFAs), and to an increased production of proinflammatory mediators (TNF-α, IL-1ß, and Il-6). The inflammatory state of AT contributes to liver inflammation and compromises its functionality, enhancing hepatic de novo lipogenesis and decreasing lipid ß-oxidation [5]. These alterations cause higher triglyceride (TG) synthesis, along with altered mitochondrial lipid oxidation and the production of lipotoxic intermediates in the liver. The production of these lipotoxic intermediates contributes to further hepatic inflammation and mitochondrial disfunction [6]. Indeed, impairments in this cell organelle lead to enhanced oxidative stress and to the production of ROS [6].

In addition, the state of the gastrointestinal tract is a key factor in NAFLD pathogenesis. In this regard, changes taking place in gut microbiota composition, namely dysbiosis (decreased bacterial diversity and richness, along with impaired gut barrier function and bacterial metabolite production), are crucial in this process. In this regard, microbiota-derived ethanol and its related metabolites (including acetaldehyde and acetate) contribute to changes in both enterocyte morphology and functionality (promoting endotoxin infiltration) and, therefore, to a rapid generation of ROS. Moreover, acetate represents a substrate for FA synthesis, which leads to increased circulating FFA levels [11]. This state of enhanced oxidative stress and inflammation in the liver contributes to hepatocyte death (apoptosis), which has been described as an additional “hit” influencing the progression of steatohepatitis towards more harmful stages [12].

Since the medical management of NAFLD is limited, the scientific community has been searching for alternative or complementary therapies that may be potentially beneficial for its amelioration or prevention. Given the aforementioned relationship between gut microbiota dysbiosis and inflammation, particular attention has been paid to treatments focused on reestablishing gut microbiota composition [13]. On this subject, probiotics have emerged as an interesting therapeutic tool for NAFLD management due to their capacity to down-regulate lipid synthesis, activate lipid oxidation, downregulate pro-inflammatory pathways, and modulate microbiota composition in animal models [14]. Although proven effective, probiotic-based therapies have some limitations. According to the Food and Agriculture Organization/World Health Organization (FAO/WHO), probiotics may predispose some individuals to certain side effects such as systemic infections, deleterious metabolic activities, excessive immune stimulation, and gene transfer [15]. This, coupled with the difficulty to maintain the viability and safety of microorganisms during their storage and industrial processing, has increased the interest in treatments based on inactivated bacteria. Consequently, parabiotics, which are nonviable microbial cells (intact or broken) or crude cell extracts that may confer benefits when consumed in enough quantities [16], have emerged as plausible alternative to overcome the limitations of probiotics. 

In this context, the current research aimed to compare the effects of a probiotic (*Lactobacillus rhamnosus* GG), and its parabiotic, on oxidative stress, inflammation, DNA damage, and cell death pathways in the liver of rats featuring liver steatosis induced by a high-fat and high-fructose diet. 

## 2. Materials and Methods

### 2.1. Animals, Diets, and Experimental Design 

A total of thirty-four (8–9-week-old) male Wistar rats (Envigo, Barcelona, Spain) were used to perform the experiment, which took place in accordance with the institution’s guide for the care and use of laboratory animals (M20/2021/214). Rats were housed in polycarbonate conventional cages (two rats per cage) and placed in an air-conditioned room (22 ± 2 °C) with a 12 h light–dark cycle. After six days of adaptation, animals were distributed into four different experimental groups. The first group of rats was fed a standard laboratory diet (C group; n = 8) (D10012G; Research Diets, New Brunswick, NJ, USA). The remaining three groups of animals were fed a high-fat high-fructose diet (D21052401; Research Diets, New Brunswick, NJ, USA) (Table 1). The rats fed the obesogenic diet received either the diet alone (HFHF group; n = 8) or supplemented with a commercially acquired viable probiotic (*Lactobacillus rhamnosus* GG, Ferring Pharmaceuticals, Switzerland) (PRO group; n = 9) or the same probiotic as a parabiotic (*Lactobacillus rhamnosus* GG heat-inactivated) (PAR group; n = 9). The probiotic was diluted in phosphate-buffered saline (PBS) containing 5% of sucrose, ensuring a dose of 10^9^ CFU/day as described elsewhere [17,18]. In the case of the parabiotic, which was inactivated by heat treatment (80 °C for 20 min), as explained elsewhere [19,20], the same probiotic- and sucrose-containing PBS dilution was used (ensuring a dose of 10^9^ CFU/day). The animals in the C and the HFHF groups received sucrose-enriched PBS as the vehicle. These experimental conditions were maintained for six weeks, an experimental period length that was selected according to the experimental conditions used by other authors to test the effects of probiotic administration in the management of NAFLD [14]. All the treatments were administered daily via oral gavage. Animals had free access to food and water, and body weight and food intake were measured on a daily basis. 

At the end of the experimental period (6 weeks), the animals were anesthetized (chloral hydrate) and euthanized after fasting (8–12 h) via cardiac exsanguination using a sterile syringe and needles. Following sacrifice, the livers were dissected using tweezers and scissors, weighed, and immediately frozen in liquid nitrogen. Blood samples were centrifuged (1000× *g* for 10 min, at 4 °C) for serum extraction. All samples were stored in an ultrafreezer at −80 °C until analysis.

### 2.2. Liver Triacylglycerol Content and Serum Transaminases

Total hepatic lipids were extracted following the method described by Folch et al. [21]. The lipid extract was dissolved in isopropanol, and the triacylglycerol content was measured via spectrophotometry using a commercial kit (Spinreact, Barcelona, Spain). As for the assessment of serum alanine aminotransferase (ALT) and aspartate aminotransferase (AST) levels, commercially available kits were also used (Biosystems, Barcelona, Spain).

### 2.3. Parameters Related to Oxidative Stress in Liver

#### 2.3.1. Lipid Peroxidation Measurement

Rat liver samples were homogenized and lipid peroxidation was determined by using a commercial TBARS assay kit (Cayman Chemical, Ann Arbor, MI, USA). According to the manufacturer’s instructions, thiobarbituric acid reactive substances (TBARS) were measured as a marker for lipid peroxidation. This method is based on the reaction of malondialdehyde (MDA) and thiobarbituric acid (TBA) in an acid medium. The amount of MDA-TBA adduct was quantified in an Infinite 200Pro plate reader (Tecan, Männedorf, Zürich, Switzerland). Results were expressed as µM MDA/mg of tissue.

#### 2.3.2. Total Antioxidant Capacity Determination

Rat liver homogenates were used to analyze the total antioxidant capacity by using the commercial kit OxiSelect Oxygen Radical Antioxidant Capacity (ORAC) activity assay (Cell Biolabs, San Diego, CA, USA). Briefly, the ORAC assay was performed using fluorescein as a fluorescence probe. AAPH (2,2-azobis [2-amidinopropane] dihydrochloride) was utilized as a free radical initiator to produce peroxyl radicals. Thus, AAPH was added to the sample and the fluorescence was reordered in an Infinite 200Pro plate reader (Tecan, Männedorf, Zürich, Switzerland). Furthermore, a calibration curve was built with Trolox solution. Finally, results were calculated based upon differences in areas under the fluorescence decay curve among blank, samples and standards, and final ORAC values were expressed as µM Trolox equivalents/mg tissue.

#### 2.3.3. Determination of Nonenzymatic Antioxidant Glutathione

The glutathione colorimetric assay kit (Biovision, Milpitas, CA, USA) was used to determine the glutathione (GSH) concentrations in rat liver homogenates. This assay is based on the glutathione recycling system in the presence of GSH and the DTNB fluorophore. When DTNB is reduced, it produces a stable fluorescent product which can be detected in an Infinite 200Pro plate reader (Tecan, Männedorf, Zürich, Switzerland). Results were expressed as µg GSH/mg of tissue.

#### 2.3.4. Superoxide Dismutase Activity (SOD; EC 1.15.1.1)

Total superoxide dismutase activity in rat liver homogenates was analyzed by the SOD activity assay kit (Sigma-Aldrich, San Louis, MO, USA) according to the manufacturer’s instructions. The method is based in the generation of superoxide anions by means of the xanthine–xanthine oxidase system. Thus, the superoxide anion reduced WST-1, which was converted into WST-1 formazan and its absorbance was recorded in an Infinite 200Pro plate reader (Tecan, Männedorf, Zürich, Switzerland). In the presence of SOD, O_2_^−^ underwent a dismutation into O_2_ and H_2_O_2_, thus decreasing the WST-1 formazan formation. The SOD activity (U/mL) was calculated according to the manufacturer’s instruction using the inhibition curve.

#### 2.3.5. Catalase (CAT; EC 1.11.1.6)

Rat liver samples were homogenized with the aim of determining catalase activity according to Aebi [22]. Briefly, the reaction took place in a final volume of 250 µL containing 90 mM potassium phosphate buffer (pH 6.8) and it started with the addition of H_2_O_2_ (37.5 mM final concentration). Next, the H_2_O_2_ disappearance was measured at 240 nm spectrophotometrically. The results were expressed as nmol/min/µg of protein.

#### 2.3.6. Glutathione Peroxidase (GPx; EC 1.11.1.9)

Glutathione peroxidase activity was analyzed by measuring the H_2_O_2_ scavenging capacity using the GPx assay kit (Biovision, Milpitas, CA, USA). GSSG was formed upon the reduction of H_2_O_2_ by GPx, and it was recycled in its reduced state (GSH) by both glutathione reductase (GR) and reduced nicotinamide adenine dinucleotide phosphate (NADPH). Next, NADPH absorbance decrease was measured in an Infinite 200Pro plate reader (Tecan, Männedorf, Zürich, Switzerland), and GPx activity was expressed as GPx U/mL.mg of protein.

#### 2.3.7. Determination of Total Proteins

Total protein was spectrophotometrically quantified in lysates and homogenates at 595 nm by Bradford assay [23], using bovine serum albumin as standard.

### 2.4. Parameters Related to Inflammation in Liver

#### IL-1β and TNF-α Determination

Interleukin 1 beta (IL-1β) and tumor necrosis factor alpha (TNF-α) amounts were determined in rat liver lysates by using commercial kits (RyD Systems, Minneapolis, MO, USA and ThermoFisher, Waltham, MA, USA, respectively).

### 2.5. Parameters Related to DNA Damage and Cell Death by Immunoblotting

For Poly (ADP-ribose) polymerase (PARP), H2A histone family member X (γH2AX), ATM serine/threonine kinase (ATM), phosphorylated-ATM (Ser 1981) and beta actin (ß-actin) protein quantification, liver samples (100 mg) were homogenized in 1 mL of cellular PBS (pH 7.4), containing protease inhibitors (100 mM phenylmethylsulfonyl fluoride and 100 mM iodoacetamide). The homogenates were centrifuged at 800× *g* for 5 min at 4 °C.

Immunoblot analyses were performed by loading 60 or 80 μg of total protein from liver extracts separated by electrophoresis in either 4–15% (PARP, γH2AX and ß-actin) or 7.5% (p-ATM and ATM) SDS-polyacrylamide gels and transferred to polyvinylidene difluoride (PVDF) membranes (Merck, Darmstadt, Germany). The membranes were then blocked with 4% BSA for 1.5 h at room temperature. Afterwards, they were blotted with the appropriate antibodies, PARP (1:1000; Cell Signaling, Danvers, MA, USA), γH2AX (1:1000; Abcam, Cambridge, UK), p-ATM (1:500; Novus Biologicals, Centennial, CO, USA), ATM (1:500; Abcam, Cambridge, UK) and ß-actin (1:1000; Cell Signaling, Danvers, MA, USA) overnight at 4 °C. Subsequently, membranes were incubated with polyclonal anti-mouse (1:5000) (Santa Cruz Biotech, Dallas, TX, USA) for γH2AX, p-ATM and ß-actin, and anti-rabbit (1:5000) (Santa Cruz Biotech, Dallas, TX, USA) for PARP and ATM, for 2 h at room temperature. The bound antibodies were visualized by an ECL system (Thermo Fisher Scientific Inc., Rockford, IL, USA) and quantified by a ChemiDoc MP Imaging System (Bio-Rad, Hercules, CA, USA). The measurements were normalized either by ß-actin or the phosphorylated isoform.

### 2.6. Statistical Analysis

Results are presented as mean ± SEM. Statistical analysis was performed using SPSS 24.0 (SPSS, Chicago, IL, USA). The normal distribution of data was assessed by Shapiro–Wilks test. Normally distributed parameters were analyzed by one-way ANOVA followed by the Newman–Keuls post hoc test. Significance was assessed at the *p* < 0.05 level.

## 3. Results

### 3.1. Body and Liver Weights, Hepatic Triglyceride Content and Serum Transaminase Levels

At the end of the experimental period, the high-fat high-fructose diet feeding resulted in a significant increase in final body weight, compared to the control group. Despite not finding significant changes regarding this parameter in the animals fed the same steatotic diet and supplemented with the probiotic or the parabiotic, a partial reduction in final body weight was observed. Indeed, trends towards reduced final body weights were found in both groups (*p* < 0.1 for PRO and *p* < 0.09 for PARA) compared to the HFHF group (Table 2). As for liver weight, a similar pattern was appreciated. In this regard, all groups fed the high-fat high-fructose diet had a significant increase in liver weight compared to the C group. As opposed to the HFHF group, nonsignificant trends towards lower values were found in the groups fed the steatotic diet and supplemented with the probiotic and the parabiotic (*p* < 0.1 for PRO and *p* < 0.07 for PARA) (Table 2).

Regarding hepatic TG content, the animals fed the high-fat high-fructose diet showed a significant increase in this parameter in comparison to the animals fed the control diet, showing that liver steatosis was achieved. The administration of the probiotic significantly reverted this effect in comparison to the nontreated animals fed the high-fat high-fructose diet (*p* < 0.05). However, the reduction in hepatic TG content found in the animals in the PRO group did not reach that observed in the C group. As for the group receiving the parabiotic, no change in this parameter was found compared to the HFHF group (Table 2). As far as serum transaminases were concerned, both ALT and AST levels were significantly increased in the HFHF group compared to the C group. In the case of ALT, none of the tested treatments resulted in significant changes in comparison with the nontreated animals fed the steatotic diet. Despite not being statistically significant, it is worth mentioning that the reductions in serum ALT levels found in the PRO and PARA groups were 23.4% and 40.1% lower than those found in the HFHF group (respectively). A similar pattern was further observed with regard to serum AST levels. Additionally, in this case, the reductions found in the PRO and PARA groups did not reach statistical significance when compared to the HFHF group. However, the administration of the probiotic and the parabiotic resulted in reductions of 44.2% and 39.8% (respectively) in this parameter compared to the HFHF group.

### 3.2. Parameters Related to Oxidative Stress in Liver

**Figure 1:**
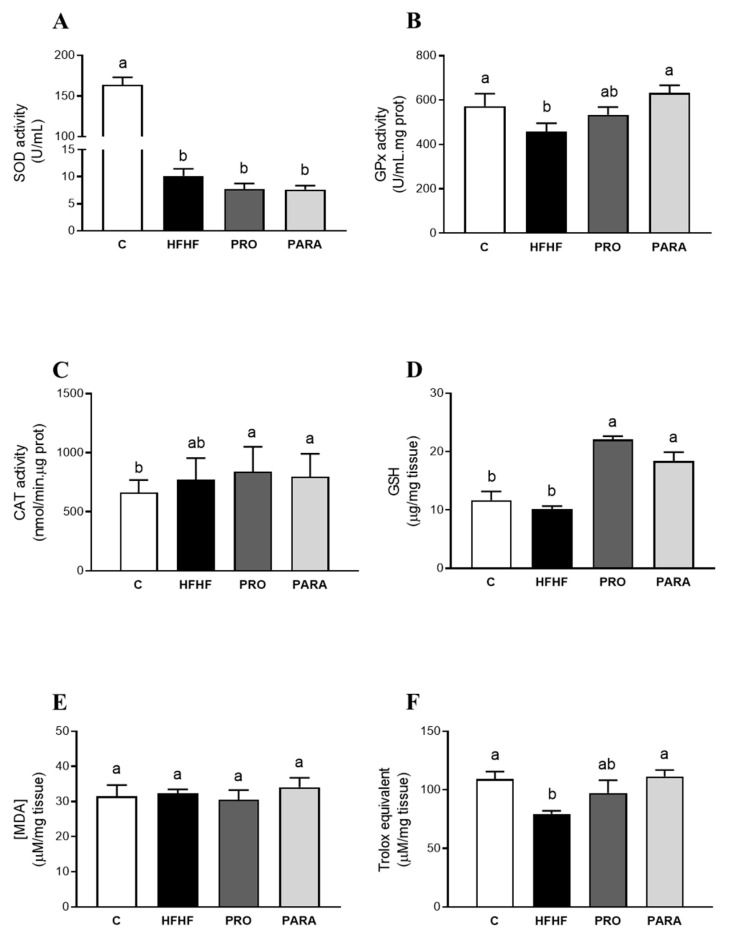
Activity of SOD (**A**), GPx (**B**) and CAT (**C**), and levels of GSH (**D**), MDA (**E**) and Trolox equivalent (**F**) in liver samples of rats fed a control diet (C), a high-fat high-fructose diet (HFHF) or a high-fat high-fructose diet supplemented with viable or heat-inactivated 10^9^ CFU/day of *Lactobacillus rhamnosus* GG (PRO and PARA, respectively). Values are presented as mean ± SEM. Differences among the groups were determined by using one-way ANOVA, followed by the Newman–Keuls post hoc test. Bars not sharing common letters are significantly different (*p* < 0.05). CAT: catalase, GPx: glutathione peroxidase, GSH: total glutathione, MDA: malondialdehyde, SOD: superoxide dismutase.

To evaluate the effects of the treatments on the hepatic oxidative stress of the animals exposed to the high-fat high-fructose diet, we measured the antioxidant capacity of the treatments and the activity of key enzymes that contribute to redox homeostasis maintenance, as well as the content of nonprotein antioxidants and lipid peroxidation products. When the activity of antioxidant enzymes was analyzed, a significant reduction in superoxide dismutase (SOD) activity, along with a sharp decrease in glutathione peroxidase (GPx) activity, was found in the nontreated animals fed the high-fat high-fructose diet, compared to the control rats (*p* < 0.001 and *p* = 0.057 vs. control group, respectively) as shown in Figure 1. A tendency to higher catalase (CAT) activity was also observed in the animals of the HFHF group compared to the control group (*p* = 0.090). Concerning nonenzymatic antioxidants, there were no differences in total glutathione (tGSH; Figure 1) levels in the HFHF group, when compared to the C group (*p* = 0.18). As for lipid peroxidation product assessment, no changes in MDA content were found in the nontreated animals fed the HFHF diet compared to the animals in the C group. Nonetheless, the total antioxidant capacity (ORAC), showed a decrease, compared to the C group (*p* < 0.001).

Concerning the antioxidant enzyme activities, the treatments were not able to restore the SOD activity decrease found in the HFHF group. Nevertheless, a nonsignificant trend towards a higher GPx activity and a significant increase were observed in the groups treated with the probiotic and the parabiotic, respectively (*p* = 0.082; *p* < 0.01). Lastly, the hepatic CAT activity was not significantly modified by the high-fat high-fructose feeding. The groups supplemented with the probiotic or the parabiotic did not show significant differences with the HFHF group but displayed significant increases with regard to the control group (*p* < 0.05 and *p* < 0.01, respectively). Concerning the nonenzymatic antioxidant protection, both the probiotic and the parabiotic administration significantly increased the GSH content compared to the HFHF group (*p* < 0.001). Indeed, the values were significantly higher than those in the C group (*p* < 0.001 and *p* < 0.01 for PRO and PARA vs. C, respectively). Regarding the effects of the treatments on lipid peroxidation, no changes in MDA content were found neither in the PRO nor in the PARA group, compared to the animals in the C and the HFHF groups. As for the antioxidant capacity, the group fed the high-fat high-fructose diet and supplemented with the probiotic showed a nonsignificant trend towards higher values compared to the HFHF group (*p* = 0.068). In the case of the animals supplemented with the parabiotic, the sharp decrease in the antioxidant capacity derived from the high-fat high-fructose diet feeding was totally reverted (*p* < 0.05 vs. HFHF).

### 3.3. Parameters Related to Inflammation in Liver

**Figure 2:**
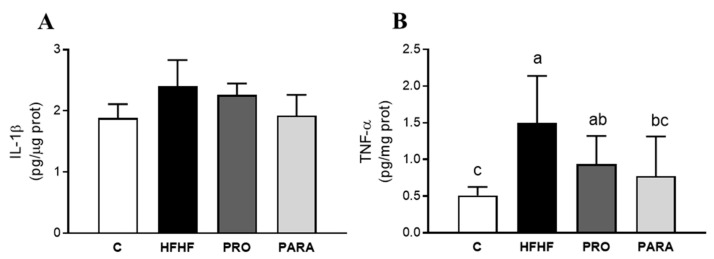
Hepatic levels of IL-1ß (**A**) and TNF-α (**B**) in rats fed a control diet (C), a high-fat high-fructose diet (HFHF) or a high-fat high-fructose diet supplemented with viable or heat-inactivated 10^9^ CFU/day of *Lactobacillus rhamnosus* GG (PRO and PARA, respectively). Values are presented as mean ± SEM. Differences among the groups were determined by using one-way ANOVA, followed by the Newman–Keuls post hoc test. Bars not sharing common letters are significantly different (*p* < 0.05). IL-1ß: interleukin 1ß, TNF-α: tumor necrosis factor α.

Due to the key role of inflammation in the pathogenesis and progression of NAFLD, the hepatic levels of IL-1β and TNF-α, two well-known inflammatory markers, were analyzed. In this regard, 1L-1β levels increased by 20% in the animals fed the high-fat high-fructose diet, compared to the control group, although this change did not reach statistical significance. Nonetheless, a sharp increase in TNF-α levels was observed in the HFHF group compared to the control animals (Figure 2).

As for the effects of the treatments, in both cases, the IL-1ß levels were similar to those found in the C and HFHF groups. In the case of TNF-α levels, animals treated with the probiotic showed a sharp but nonsignificant decrease in the hepatic levels of this cytokine compared to the HFHF group (*p* = 0.075). Lastly, the parabiotic-treated animals displayed significantly lower hepatic TNF-α content compared to the HFHF group (*p* < 0.05), reaching values similar to those found in the C group.

### 3.4. Parameters Related to DNA Damage and Cell Death by Immunoblotting

**Figure 3:**
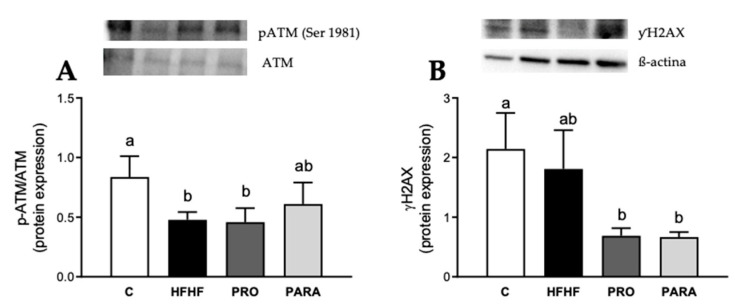
Activation rate (phosphorylation) of ATM (**A**) and protein expression levels of γH2AX (**B**) in liver samples of rats fed a control diet (C), a high-fat high-fructose diet (HFHF) or a high-fat high-fructose diet supplemented with viable or heat-inactivated 10^9^ CFU/day of *Lactobacillus rhamnosus* GG (PRO and PARA, respectively). Values are presented as mean ± SEM. Differences among the groups were determined by using one-way ANOVA, followed by the Newman–Keuls post hoc test. Bars not sharing common letters are significantly different (*p* < 0.05). ATM: ataxia-telangiectasia mutated, γH2AX: phosphorylated histone H2AX.

Owing to the harmful effects that both oxidative stress and inflammation have on DNA nature and cell viability, some of the key molecular markers of these processes were also studied. In this scenario, a very early response of mammalian cells to DNA double-strand breaks is the phosphorylation of histone H2AX at serine 139 (γH2AX), at the sites of DNA damage by the protein kinase ataxia-telangiectasia mutated (ATM). Firstly, the high-fat high-fructose feeding induced a reduction in the activation rate of ATM (p-ATM/ATM ratio) compared to the C group (*p* = 0.044) (Figure 3). By contrast, regarding the expression of γH2AX, no significant changes were observed between these same experimental groups.

Concerning the effects of the treatments on the aforementioned parameters, no differences were observed in ATM activation with either the probiotic or the parabiotic compared to the HFHF group. Regarding γH2AX protein expression levels, a reduction was noted in both treated groups compared to the HFHF group (−61.90% PRO; −63.20% PARA). These differences showed a statistical trend (*p* = 0.070 PRO vs. HFHF; *p* = 0.066 PARA vs. HFHF) that reached statistical significance when compared to the C group (*p* < 0.05 PRO vs. C; *p* < 0.05 PARA vs. C).

**Figure 4:**
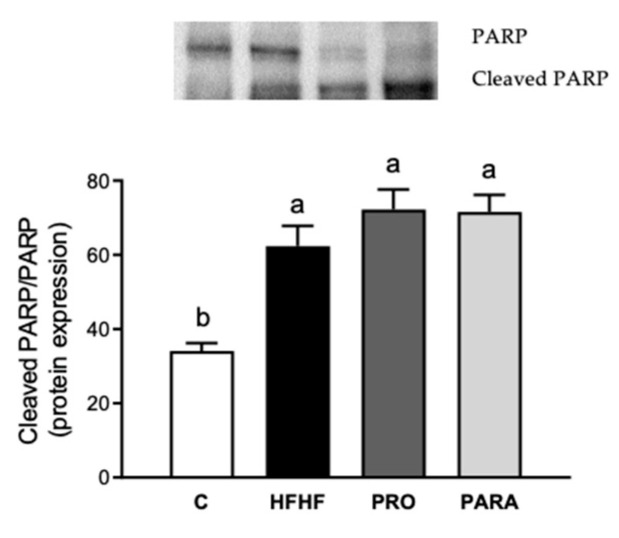
Cleavage rate of PARP in liver samples of rats fed a control diet (C), a high-fat high-fructose diet (HFHF) or a high-fat high-fructose diet supplemented with viable or heat-inactivated 10^9^ CFU/day of *Lactobacillus rhamnosus* GG (PRO and PARA, respectively). Values are presented as mean ± SEM. Differences among the groups were determined by using one-way ANOVA, followed by the Newman–Keuls post hoc test. Bars not sharing common letters are significantly different (*p* < 0.05). PARP: poly (ADP-ribose) polymerase.

Regarding cell-death-related markers, the high-fat high-fructose diet-induced a significant increase in the protein expression of the cleaved fraction of PARP in comparison to the C group (*p* = 0.001). This effect was not reverted by either the probiotic or the parabiotic treatment (Figure 4).

## 4. Discussion

Unbalanced dietary patterns have been identified as a major contributor to the development of obesity and related metabolic alterations. In this regard, the consumption of diets rich in saturated fats and sugars (such as fructose) is known to impair glucose homeostasis and lipid metabolism, leading to the development of insulin resistance and NAFLD [24].

In the present study, feeding a diet rich in saturated fat and fructose to rats led to increased body and liver weight, along with a greater hepatic TG content, proving to be a dietary pattern effective to generate a model of steatosis. According to the reported results, the administration of a viable probiotic, as well as its heat-inactivated parabiotic, resulted in a slight decrease in both parameters (nonsignificant trends towards lower values). These results are in line with the available literature, in which the effectiveness of probiotic administration has been described for the management of obesity in both murine models and humans [25,26,27]. The lack of statistical significance obtained in the present study regarding these parameters may well be due to the selected experimental length, model and/or the probiotic strain. As for the parabiotic, similar results have been reported in a recently published study, despite the used experimental model (male C57BL/6 J mice), probiotic strain (*Lactiplantibacillus plantarum* K8) and experimental period length (14 weeks) being different [28]. Regarding the hepatic TG content, the administration of the probiotic resulted in a significant decrease in this parameter, which is in good accordance with the data published to date and reviewed elsewhere [14]. By contrast, the administration of the parabiotic did not ameliorate the liver TG accumulation induced by the high-fat high-fructose diet. Notwithstanding the scarcity of the available studies addressing the effects of parabiotics on hepatic lipid accumulation, it has been reported that the administration of an inactivated mixture of lactoferrin-expressing probiotic strains resulted in a lowered hepatic lipid content in mice fed a high-fat diet [29]. In spite of this apparent discrepancy, it must be noted that the experimental design used in that study differs significantly from that used in the present research.

Serum transaminase levels are another indicator that is commonly assessed when studying liver damage, as well as the potential benefits induced by the administration of bioactives. In this regard, the high-fat high-fructose diet caused a significant increase in the serum levels of ALT and AST, which is in good accordance with the vast majority of studies using similar experimental models [30,31,32]. In this case, neither the administration of the probiotic nor the parabiotic resulted in significant changes in these parameters, despite finding sharp reductions in both transaminase levels. These results are in accordance with those reported by Zhao et al. [33]. Moreover, several studies researching the potential usefulness of probiotics for NAFLD management have not reported changes in serum transaminase levels despite other benefits (such as decreased liver triglyceride content) being described [14].

Besides excessive hepatic lipid accumulation, alterations in the electron flow of the respiratory chain, leading to the formation of reactive oxygen species (ROS), also occur during the steatotic process [34], which result in an imbalance between ROS and antioxidant defenses in favor of the former [35,36]. Thus, some parameters related to oxidative stress were analyzed in the present study to better elucidate the potential benefits of probiotic and parabiotic administration in diet-induced NAFLD management.

The results show that the ability of the antioxidant enzymes SOD and GPx to neutralize ROS diminished in rats under the high-fat high-fructose diet, which is in good accordance with the reduction observed in the antioxidant capacity, measured by ORAC. However, the CAT enzyme activity, which catalyzes the decomposition of hydrogen peroxide to water and oxygen, and the nonenzymatic antioxidant GSH levels were not significantly modified in rats fed the steatotic diet. Due to the alteration of both SOD and GPx enzymatic activities induced by the high-fat high-fructose diet, a rise in superoxide anion and hydrogen peroxide production could be expected in this experimental model, which may induce a detrimental oxidative status, even though the oxidative stress of lipids was still rather nonexistent. Although controversial results have been reported with regard to changes in the antioxidant enzyme system observed in NAFLD models [37,38], the results reported in this study are in good accordance with those shown in several studies of rats fed a high-fat high-fructose diet [39,40,41,42]. In fact, in a rat model of obesity, steatosis and insulin resistance induced by a high-fat high-fructose diet, a down-regulation of SOD, GPx and hepatic total GSH content prompted an imbalance in the oxidant/antioxidant system [43]. Moreover, Feillet-Coudray et al. [43] argued that lipid peroxidation also remained very low after 20 weeks of high-fat high-fructose diet feeding. Thus, the authors argued that hepatic lipid accumulation induces moderate oxidative stress which is not sufficient to cause a significant MDA accumulation in the livers of animals under a high-fat high-fructose diet despite a marked oxidative status appearing at the end of the steatotic stage.

It is well known that probiotics play a key role as free radical scavengers, being able to reduce the damage caused by oxidation [44,45,46]. In addition, parabiotics also exhibit antioxidant activity, but the available literature that supports this is still scarce [47]. In the present study, when rats under high-fat high-fructose feeding were supplemented with a commercially acquired viable probiotic (*Lactobacillus rhamnosus* GG), this bacterium effectively increased hepatic GSH levels compared to the HFHF group. Moreover, probiotic administration was able to partially prevent (*p* = 0.08) the HFHF diet-induced GPx depletion. These effects were also observed when the selected probiotic was administered after heat inactivation (parabiotic). In this line, it was observed that the parabiotic was able to completely restore the down-regulation induced in GPx activity and the depletion of antioxidant capacity prompted by the high-fat high-fructose diet. In addition, the nonviable bacteria significantly increased the nonenzymatic GSH antioxidant. Taken together, these results show that the parabiotic treatment was effective in defending the liver against the oxidative stress induced by the high-fat high-fructose diet feeding to a greater extent than the viable *Lactobacillus rhamnosus* GG. The results obtained with the probiotic are consistent with those reported by other authors using another strain of Lactobacillus. In this line, Park et al. [48] observed increased levels of either GPx or CAT activity in rats fed a high-fat high-fructose diet supplemented with two *Lactobacillus plantarum* strains for eight weeks, without restoring SOD inhibition induced by the HFHF diet. Thus, even longer experimental periods (eight instead of six weeks, as in the present study) did not prevent the antioxidant SOD reduction induced by a high-fat high-fructose diet. It should be noted that, although there are studies focused on the effects of parabiotics on high-fat-diet-induced obese rats [49], the data reported in the present study cannot be compared to other studies, since no data concerning the effects of these inactivated bacteria on oxidative status have been reported so far.

Hepatic inflammation is a key component in the progression of NAFLD to steatohepatitis [50,51]. In fact, the lipotoxicity induced by fatty hepatocytes contributes to the activation of Kupffer cells, which produce inflammatory cytokines such as IL-1β and TNF-α, two crucial inflammation markers and relevant mediators in the development of NAFLD [52]. In this cohort of rats, although IL-1ß levels in the HFHF group were higher than in the control group, statistical significance was not reached. Regarding TNF-α, the steatotic diet induced a two-fold increase that was partially prevented by the probiotic and completely restored to control values by the parabiotic. As in the case of oxidative stress, both the probiotic and the parabiotic were efficient in preventing inflammation, but the latter seemed to be more effective.

A relevant issue to consider regarding the NAFLD pathophysiology is that this metabolic disorder contributes to the DNA damage and, thus, can favor the development of hepatocellular carcinoma [53,54]. To address this issue, the protein kinase ATM protein, which is activated by double-strand DNA breaks (DSB), was measured. Once activated, ATM activates intermediary protein substrates, such as γH2AX, leading to cell cycle arrest triggering DNA repair. In the present study, it was observed that the high-fat high-fructose diet significantly decreased ATM phosphorylation, although no changes were observed in the DNA damage marker γH2AX. These results are in good accordance with those reported by Daugherity et al. [55], who observed a significantly increased oxidative stress in the liver of high-fat diet-fed mice (eight weeks), without substantial activation of H2AX protein. In addition, other authors have also reported decreased p-ATM levels in nonhepatic tissues (skeletal muscle) in mice fed a high-fat diet for eight weeks [56]. Nevertheless, it is important to consider that other kinases, such as Ataxia-telangiectasia and Rad3-related protein (ATR) and DNA-dependent protein kinase (DNA-PK), play a key role in the phosphorylation of H2AX protein in the response to DNA injury. Overall, these data suggest that, although high-fat high-fructose feeding promotes a significant oxidative status, it does not induce nucleic acid damage in a period feeding of 6–8 weeks.

In order to assess the relationship between inflammation and cell death, and taking into account that the TNF-α overexpression could activate proapoptotic pathways in the rats under the high-fat high-fructose diet, PARP cleavage was studied since this protein is a preferred substrate for caspases [57]. The obtained results show that, compared to the control group, the steatotic diet up-regulated PARP cleavage which is essential for cell death signaling. It is noteworthy that apoptotic hepatocytes stimulate hepatic stellate cells and immune cell activation, both being central to the progression of NAFLD and steatohepatitis [58]. However, none of the tested treatments (probiotic or parabiotic administration) significantly avoided this effect. This suggests that the dietary model used in the present study activated apoptosis, albeit treatments were not able to prevent this situation, and thus, they seem not to be effective preventing the progression of hepatic damage towards more harmful stages through this pathway. In fact, Karahan et al. [59] reported that the modulation of apoptosis in a nonalcoholic steatohepatitis model of rats supplemented with a probiotic was different depending on the experimental period or the probiotic administration. Therefore, one limitation of the present study is that the chosen experimental period (6-weeks) seems to be insufficient for prompting DNA breaks and modulating hepatocyte cell death. For this reason, further research with longer experimental periods is needed in order to determine whether the treated animals would gain more benefits from the probiotic and parabiotic, fighting against the hepatotoxicity induced by the steatotic diet.

To sum up, the current results show that the probiotic *Lactobacillus rhamnosus* GG administration is able to partially prevent the oxidative stress and the inflammation induced in the liver by high-fat high-fructose feeding. Moreover, it is demonstrated for the first time that the parabiotic (heat-inactivated *Lactobacillus rhamnosus* GG) maintains both antioxidative and anti-inflammatory activities, and it seems to be even more effective than the originating probiotic, avoiding the potential side effects of the viable bacteria such as systemic infections and excessive immune stimulation. Although bacterial viability and cell wall integrity have been thought to be indispensable requisites for probiotic action, some of the bacterial molecule’s key in host metabolism regulation are located within bacteria [60]. In this regard, several authors highlighted the potential role of lysozyme, which disrupts bacterial membranes and releases bacterial products with beneficial effects on the host, such as anti-inflammatory effects in mucosal sites [61]. This could partially explain why the effectiveness of the supplementation with already-inactivated microorganisms seems to be greater in comparison with its viable counterpart.

## Figures and Tables

**Table 1:** Nutrient composition of experimental diets.

	STD	HFHF
Total energy (kcal/g)	3.9	4.5
Composition by energy%		
Carbohydrates	63.9	40
Fructose	-	10
Proteins	20.3	20
Lipids	15.8	40

g: grams, HFHF: high-fat high-fructose, kcal: kilocalories, STD: standard diet.

**Table 2:** Final body weight, food intake, liver weight, hepatic triacylglycerol (TG) content, serum alanine aminotransferase (ALT) level and aspartate aminotransferase (AST) level of rats fed a standard diet (C) and a high-fat high-fructose diet alone (HFHF) or supplemented with viable or heat-inactivated *Lactobacillus rhamnosus* GG (PRO and PARA, respectively) for six weeks.

	C	HFHF	PRO	PARA	ANOVA
Final body weight (g)	407 ± 14 ^b^	469 ± 14 ^a^	435 ± 14 ^ab^	436 ± 12 ^ab^	*p* < 0.01
Food intake (g)	20.3 ± 0.5	21.4 ± 0.4	20.2 ± 0.6	20.3 ± 0.5	NS
Liver weight (g)	11.8 ± 1.0 ^b^	21.5 ± 1.2 ^a^	19.0 ± 0.9 ^a^	18.8 ± 0.7 ^a^	*p* < 0.001
Liver TG (mg/g tissue)	42.7 ± 1.7 ^c^	130.5 ± 4.6 ^a^	108.2 ± 4.4 ^b^	119.4 ± 4.6 ^ab^	*p* < 0.05
ALT (U/L)	13.6 ± 3.1 ^b^	46.6 ± 11.5 ^a^	32.3 ± 2.6 ^a^	27.9 ± 3.2 ^a^	*p* < 0.05
AST (U/L)	43.9 ± 1.8 ^b^	101.3 ± 21.6 ^a^	56.5 ± 6.5 ^ab^	61.0 ± 5.0 ^a^	*p* < 0.05

Values are presented as mean ± SEM. Differences among groups were determined by using a one-way ANOVA followed by the Newman–Keuls post hoc test. Values not sharing a common letter are significantly different (*p* < 0.05). NS: not significant.

## Data Availability

Not applicable.

## References

[1] Riazi K., Azhari H., Charette J.H., Underwood F.E., King J.A., Afshar E.E., Swain M.G., Congly S.E., Kaplan G.G., Shaheen A.A. (2022). The Prevalence and Incidence of NAFLD Worldwide: A Systematic Review and Meta-Analysis. Lancet Gastroenterol. Hepatol..

[2] Reyes-Gordillo K., Shah R., Muriel P. (2017). Oxidative Stress and Inflammation in Hepatic Diseases: Current and Future Therapy. Oxid. Med. Cell. Longev..

[3] Hong T., Chen Y., Li X., Lu Y. (2021). The Role and Mechanism of Oxidative Stress and Nuclear Receptors in the Development of NAFLD. Oxid. Med. Cell. Longev..

[4] Alsaif F., Al-hamoudi W., Alotaiby M., Alsadoon A., Almayouf M., Almadany H., Abuhaimed J., Ghufran N., Merajuddin A., Ali Khan I. (2022). Molecular Screening via Sanger Sequencing of the Genetic Variants in Non-Alcoholic Fatty Liver Disease Subjects in the Saudi Population: A Hospital-Based Study. Metabolites.

[5] Tilg H., Adolph T.E., Moschen A.R. (2021). Multiple Parallel Hits Hypothesis in Nonalcoholic Fatty Liver Disease: Revisited After a Decade. Hepatology.

[6] Buzzetti E., Pinzani M., Tsochatzis E.A. (2016). The Multiple-Hit Pathogenesis of Non-Alcoholic Fatty Liver Disease (NAFLD). Metabolism.

[7] Godoy-Matos A.F., Silva Júnior W.S., Valerio C.M. (2020). NAFLD as a Continuum: From Obesity to Metabolic Syndrome and Diabetes. Diabetol. Metab. Syndr..

[8] Softic S., Cohen D.E., Kahn C.R. (2016). Role of Dietary Fructose and Hepatic De Novo Lipogenesis in Fatty Liver Disease. Dig. Dis. Sci..

[9] Inci M.K., Park S.-H., Helsley R.N., Attia S.L., Softic S. (2022). Fructose Impairs Fat Oxidation: Implications for the Mechanism of Western Diet-Induced NAFLD. J. Nutr. Biochem..

[10] Jensen T., Abdelmalek M.F., Sullivan S., Nadeau K.J., Green M., Roncal C., Nakagawa T., Kuwabara M., Sato Y., Kang D.H. (2018). Fructose and Sugar: A Major Mediator of Non-Alcoholic Fatty Liver Disease. J. Hepatol..

[11] Borrelli A., Bonelli P., Tuccillo F.M., Goldfine I.D., Evans J.L., Buonaguro F.M., Mancini A. (2018). Role of Gut Microbiota and Oxidative Stress in the Progression of Non-Alcoholic Fatty Liver Disease to Hepatocarcinoma: Current and Innovative Therapeutic Approaches. Redox Biol..

[12] Machado M.V., Cortez-Pinto H. (2011). Cell Death and Nonalcoholic Steatohepatitis: Where Is Ballooning Relevant?. Expert Rev. Gastroenterol. Hepatol..

[13] Briskey D., Heritage M., Jaskowski L.A., Peake J., Gobe G., Subramaniam V.N., Crawford D., Campbell C., Vitetta L. (2016). Probiotics Modify Tight-Junction Proteins in an Animal Model of Nonalcoholic Fatty Liver Disease. Therap. Adv. Gastroenterol..

[14] Arellano-García L., Portillo M.P., Martínez J.A., Milton-Laskibar I. (2022). Usefulness of Probiotics in the Management of NAFLD: Evidence and Involved Mechanisms of Action from Preclinical and Human Models. Int. J. Mol. Sci..

[15] Doron S., Snydman D.R. (2015). Risk and Safety of Probiotics. Clin. Infect. Dis..

[16] Nataraj B.H., Ali S.A., Behare P.V., Yadav H. (2020). Postbiotics-Parabiotics: The New Horizons in Microbial Biotherapy and Functional Foods. Microb. Cell Fact..

[17] Cheng Y.C., Liu J.R. (2020). Effect of *Lactobacillus rhamnosus* GG on Energy Metabolism, Leptin Resistance, and Gut Microbiota in Mice with Diet-Induced Obesity. Nutrients.

[18] Owens J.A., Saeedi B.J., Naudin C.R., Hunter-Chang S., Barbian M.E., Eboka R.U., Askew L., Darby T.M., Robinson B.S., Jones R.M. (2021). *Lactobacillus rhamnosus* GG Orchestrates an Antitumor Immune Response. Cell. Mol. Gastroenterol. Hepatol..

[19] Li N., Russell W.M., Douglas-Escobar M., Hauser N., Lopez M., Neu J. (2009). Live and Heat-Killed *Lactobacillus rhamnosus* GG: Effects on Proinflammatory and Anti-Inflammatory Cytokines/Chemokines in Gastrostomy-Fed Infant Rats. Pediatr. Res..

[20] Zhang L., Li N., Caicedo R., Neu J. (2005). Alive and Dead *Lactobacillus rhamnosus* GG Decrease Tumor Necrosis Factor-Alpha-Induced Interleukin-8 Production in Caco-2 Cells. J. Nutr..

[21] Folch J., Lees M., Sloane Stanley G.H. (1957). A Simple Method for the Isolation and Purification of Total Lipides from Animal Tissues. J. Biol. Chem..

[22] Aebi H. (1984). Catalase in Vitro. Methods Enzymol..

[23] Bradford M.M. (1976). A Rapid and Sensitive Method for the Quantitation of Microgram Quantities of Protein Utilizing the Principle of Protein-Dye Binding. Anal. Biochem..

[24] Perumpail B.J., Cholankeril R., Yoo E.R., Kim D., Ahmed A. (2017). An Overview of Dietary Interventions and Strategies to Optimize the Management of Non-Alcoholic Fatty Liver Disease. Diseases.

[25] Wang M., Zhang B., Hu J., Nie S., Xiong T., Xie M. (2020). Intervention of Five Strains of *Lactobacillus* on Obesity in Mice Induced by High-Fat Diet. J. Funct. Foods.

[26] Soundharrajan I., Kuppusamy P., Srisesharam S., Lee J.C., Sivanesan R., Kim D., Choi K.C. (2020). Positive Metabolic Effects of Selected Probiotic Bacteria on Diet-Induced Obesity in Mice Are Associated with Improvement of Dysbiotic Gut Microbiota. FASEB J..

[27] Abenavoli L., Scarpellini E., Colica C., Boccuto L., Salehi B., Sharifi-Rad J., Aiello V., Romano B., de Lorenzo A., Izzo A.A. (2019). Gut Microbiota and Obesity: A Role for Probiotics. Nutrients.

[28] Lim J.J., Jung A.H., Joo Suh H., Choi H.S., Kim H. (2022). *Lactiplantibacillus plantarum* K8-Based Paraprobiotics Prevents Obesity and Obesity-Induced Inflammatory Responses in High Fat Diet-Fed Mice. Food Res. Int..

[29] Liu Z.-S., Li P.-L., Ku Y.-W., Chen P.-W. (2022). Oral Administration of Recombinant Lactoferrin-Expressing Probiotics Ameliorates Diet-Induced Lipid Accumulation and Inflammation in Non-Alcoholic Fatty Liver Disease in Mice. Microorganisms.

[30] Chen X.X., Xu Y.Y., Wu R., Chen Z., Fang K., Han Y.X., Yu Y., Huang L.L., Peng L., Ge J.F. (2019). Resveratrol Reduces Glucolipid Metabolic Dysfunction and Learning and Memory Impairment in a NAFLD Rat Model: Involvement in Regulating the Imbalance of Nesfatin-1 Abundance and Copine 6 Expression. Front. Endocrinol..

[31] Huang Y., Lang H., Chen K., Zhang Y., Gao Y., Ran L., Yi L., Mi M., Zhang Q. (2020). Resveratrol Protects against Nonalcoholic Fatty Liver Disease by Improving Lipid Metabolism and Redox Homeostasis via the PPARα Pathway. Appl. Physiol. Nutr. Metab..

[32] Yan Y., Liu C., Zhao S., Wang X., Wang J., Zhang H., Wang Y., Zhao G. (2020). Probiotic *Bifidobacterium lactis* V9 Attenuates Hepatic Steatosis and Inflammation in Rats with Non-Alcoholic Fatty Liver Disease. AMB Express.

[33] Zhao C., Liu L., Liu Q., Li F., Zhang L., Zhu F., Shao T., Barve S., Chen Y., Li X. (2019). Fibroblast Growth Factor 21 Is Required for the Therapeutic Effects of *Lactobacillus rhamnosus* GG against Fructose-Induced Fatty Liver in Mice. Mol. Metab..

[34] Sanyal A.J., Campbell-Sargent C., Mirshahi F., Rizzo W.B., Contos M.J., Sterling R.K., Luketic V.A., Shiffman M.L., Clore J.N. (2001). Nonalcoholic Steatohepatitis: Association of Insulin Resistance and Mitochondrial Abnormalities. Gastroenterology.

[35] Browning J.D., Horton J.D. (2004). Molecular Mediators of Hepatic Steatosis and Liver Injury. J. Clin. Investig..

[36] Auger C., Alhasawi A., Contavadoo M., Appanna V.D. (2015). Dysfunctional Mitochondrial Bioenergetics and the Pathogenesis of Hepatic Disorders. Front. Cell Dev. Biol..

[37] Yoshioka S., Hamada A., Jobu K., Yokota J., Onogawa M., Kyotani S., Miyamura M., Saibara T., Onishi S., Nishioka Y. (2010). Effects of Eriobotrya Japonica Seed Extract on Oxidative Stress in Rats with Non-Alcoholic Steatohepatitis. J. Pharm. Pharmacol..

[38] Song L., Qu D., Zhang Q., Jiang J., Zhou H., Jiang R., Li Y., Zhang Y., Yan H. (2017). Phytosterol Esters Attenuate Hepatic Steatosis in Rats with Non-Alcoholic Fatty Liver Disease Rats Fed a High-Fat Diet. Sci. Rep..

[39] Dornas W.C., de Lima W.G., dos Santos R.C., da Costa Guerra J.F., de Souza M.O., Silva M., Souza e Silva L., Diniz M.F., Silva M.E. (2013). High Dietary Salt Decreases Antioxidant Defenses in the Liver of Fructose-Fed Insulin-Resistant Rats. J. Nutr. Biochem..

[40] Martinez O.D.M., Theodoro J.M.V., Grancieri M., Toledo R.C.L., de Barros F.A.R., Tako E., Queiroz V.A.V., Martino H.S.D. (2021). Dry Heated Sorghum BRS 305 Hybrid Flour as a Source of Resistant Starch and Tannins Improves Inflammation and Oxidative Stress in Wistar Rats Fed with a High-Fat High-Fructose Diet. Food Funct..

[41] Abdelmoneim D., El-Adl M., El-Sayed G., El-Sherbini E.S. (2021). Protective Effect of Fenofibrate against High-Fat–High-Fructose Diet Induced Non-Obese NAFLD in Rats. Fundam. Clin. Pharmacol..

[42] Zhao Q., Li L., Zhu Y., Hou D., Li Y., Guo X., Wang Y., Olatunji O.J., Wan P., Gong K. (2020). Kukoamine B Ameliorate Insulin Resistance, Oxidative Stress, Inflammation and Other Metabolic Abnormalities in High-Fat/High-Fructose-Fed Rats. Diabetes Metab. Syndr. Obes..

[43] Feillet-Coudray C., Fouret G., Vigor C., Bonafos B., Jover B., Blachnio-Zabielska A., Rieusset J., Casas F., Gaillet S., Landrier J.F. (2019). Long-Term Measures of Dyslipidemia, Inflammation, and Oxidative Stress in Rats Fed a High-Fat/High-Fructose Diet. Lipids.

[44] Mishra V., Shah C., Mokashe N., Chavan R., Yadav H., Prajapati J. (2015). Probiotics as Potential Antioxidants: A Systematic Review. J. Agric. Food Chem..

[45] Wang Y., Wu Y., Wang Y., Xu H., Mei X., Yu D., Wang Y., Li W. (2017). Antioxidant Properties of Probiotic Bacteria. Nutrients.

[46] Grajek W., Olejnik A., Sip A. (2005). Probiotics, Prebiotics and Antioxidants as Functional Foods. Acta Biochim. Pol..

[47] Song M.W., Jang H.J., Kim K.T., Paik H.D. (2019). Probiotic and Antioxidant Properties of Novel *Lactobacillus. brevis.* KCCM 12203P Isolated from Kimchi and Evaluation of Immune-Stimulating Activities of Its Heat-Killed Cells in RAW 264.7 Cells. J. Microbiol. Biotechnol..

[48] Park E.J., Lee Y.S., Kim S.M., Park G.S., Lee Y.H., Jeong D.Y., Kang J., Lee H.J. (2020). Beneficial Effects *of Lactobacillus plantarum* Strains on Non-Alcoholic Fatty Liver Disease in High Fat/High Fructose Diet-Fed Rats. Nutrients.

[49] Lee J.H., Woo K.J., Kim M.A., Hong J., Kim J., Kim S.H., Han K.-l., Iwasa M., Kim T.J. (2022). Heat-Killed *Enterococcus faecalis* Prevents Adipogenesis and High Fat Diet-Induced Obesity by Inhibition of Lipid Accumulation through Inhibiting C/EBP-α; and PPAR-γ; in the Insulin Signaling Pathway. Nutrients.

[50] Shaker M.E. (2022). The Contribution of Sterile Inflammation to the Fatty Liver Disease and the Potential Therapies. Biomed. Pharmacother..

[51] Astarini F.D., Ratnasari N., Wasityastuti W. (2022). Update on Non-Alcoholic Fatty Liver Disease-Associated Single Nucleotide Polymorphisms and Their Involvement in Liver Steatosis, Inflammation, and Fibrosis: A Narrative Review. Iran. Biomed. J..

[52] Arrese M., Cabrera D., Kalergis A.M., Feldstein A.E. (2016). Innate Immunity and Inflammation in NAFLD/NASH. Dig. Dis. Sci..

[53] Golabi P., Rhea L., Henry L., Younossi Z.M. (2019). Hepatocellular Carcinoma and Non-Alcoholic Fatty Liver Disease. Hepatol. Int..

[54] Massoud O., Charlton M. (2018). Nonalcoholic Fatty Liver Disease/Nonalcoholic Steatohepatitis and Hepatocellular Carcinoma. Clin. Liver Dis..

[55] Daugherity E.K., Balmus G., al Saei A., Moore E.S., Abi Abdallah D., Rogers A.B., Weiss R.S., Maurer K.J. (2012). The DNA Damage Checkpoint Protein ATM Promotes Hepatocellular Apoptosis and Fibrosis in a Mouse Model of Non-Alcoholic Fatty Liver Disease. Cell Cycle.

[56] Zhang Y.J., Zhao H., Dong L., Zhen Y.F., Xing H.Y., Ma H.J., Song G.Y. (2019). Resveratrol Ameliorates High-Fat Diet-Induced Insulin Resistance and Fatty Acid Oxidation via ATM-AMPK Axis in Skeletal Muscle. Eur. Rev. Med. Pharmacol. Sci..

[57] Chaitanya G.V., Alexander J.S., Babu P.P. (2010). PARP-1 Cleavage Fragments: Signatures of Cell-Death Proteases in Neurodegeneration. Cell. Commun. Signal..

[58] Kanda T., Matsuoka S., Yamazaki M., Shibata T., Nirei K., Takahashi H., Kaneko T., Fujisawa M., Higuchi T., Nakamura H. (2018). Apoptosis and Non-Alcoholic Fatty Liver Diseases. World J. Gastroenterol..

[59] Karahan N., Işler M., Koyu A., Karahan A.G., Başyiǧt Kiliç G., Çiriş I.M., Sütçü R., Onaran I., Çam H., Keskin M. (2012). Effects of Probiotics on Methionine Choline Deficient Diet-Induced Steatohepatitis in Rats. Turk. J. Gastroenterol..

[60] Piqué N., Berlanga M., Miñana-Galbis D. (2019). Health Benefits of Heat-Killed (Tyndallized) Probiotics: An Overview. Int. J. Mol. Sci..

[61] Ragland S.A., Criss A.K. (2017). From Bacterial Killing to Immune Modulation: Recent Insights into the Functions of Lysozyme. PLoS Pathog..

